# The Different Potential of Sponge Bacterial Symbionts in N_2_ Release Indicated by the Phylogenetic Diversity and Abundance Analyses of Denitrification Genes, *nirK* and *nosZ*

**DOI:** 10.1371/journal.pone.0065142

**Published:** 2013-06-10

**Authors:** Xia Zhang, Liming He, Fengli Zhang, Wei Sun, Zhiyong Li

**Affiliations:** Marine Biotechnology Laboratory, State Key Laboratory of Microbial Metabolism and School of Life Sciences and Biotechnology, Shanghai Jiao Tong University, Shanghai, P.R. China; Argonne National Laboratory, United States of America

## Abstract

Nitrogen cycle is a critical biogeochemical process of the oceans. The nitrogen fixation by sponge cyanobacteria was early observed. Until recently, sponges were found to be able to release nitrogen gas. However the gene-level evidence for the role of bacterial symbionts from different species sponges in nitrogen gas release is limited. And meanwhile, the quanitative analysis of nitrogen cycle-related genes of sponge microbial symbionts is relatively lacking. The *nirK* gene encoding nitrite reductase which catalyzes soluble nitrite into gas NO and *nosZ* gene encoding nitrous oxide reductase which catalyzes N_2_O into N_2_ are two key functional genes in the complete denitrification pathway. In this study, using *nirK* and *nosZ* genes as markers, the potential of bacterial symbionts in six species of sponges in the release of N_2_ was investigated by phylogenetic analysis and real-time qPCR. As a result, totally, 2 OTUs of *nirK* and 5 OTUs of *nosZ* genes were detected by gene library-based saturated sequencing. Difference phylogenetic diversity of *nirK* and *nosZ* genes were observed at OTU level in sponges. Meanwhile, real-time qPCR analysis showed that *Xestospongia testudinaria* had the highest abundance of *nosZ* gene, while *Cinachyrella* sp. had the greatest abundance of *nirK* gene. Phylogenetic analysis showed that the *nirK* and *nosZ* genes were probably of *Alpha-, Beta-,* and *Gammaproteobacteria* origin. The results from this study suggest that the denitrification potential of bacteria varies among sponges because of the different phylogenetic diversity and relative abundance of *nosZ* and *nirK* genes in sponges. Totally, both the qualitative and quantitative analyses of *nirK* and *nosZ* genes indicated the different potential of sponge bacterial symbionts in the release of nitrogen gas.

## Introduction

The oceans are a central feature of the biosphere with biogeochemical links to atmosphere. The microorganisms in seawater maintain the fertility of the ocean by catalyzing C/N/P/S transformation reactions to provide nutrients for marine organisms. Nitrogen cycle, which is driven by complex biogeochemical transformations, including nitrogen fixation, nitrification and denitrification, and assimilation, mediated by microorganisms, is a critical biogeochemical process of the oceans because it controls the productivity of the oceans and results in production and consumption of greenhouse gases [1].

Marine sponges (*Porifera*) are thought to have evolved approximately ∼580 million years and are among the most ancient metazoans [2], [3]. They represent a significant component of the marine biosphere throughout the coral reefs and benthic ecosystem. Sponges are sessile filter feeders that pump large amount of seawater every day (*e.g.* over many thousands of litres of water per day) [4], through numerous tiny pores on their surface by the flagella motion of their choanocyte cells. The ecological functions of sponges, especially N cycle [5]–[7], have attracted much more attention to the researchers. For example, sponge *Geodia barretti* has been reported to carry out nitrification rate of 566 nmol N cm^−3^ sponge day^−1^ [5], and the majority of the benthic nitrification on the Florida Keys outer reef tract probably occurs in sponges [6]. Sponges are known to harbor phylogenetically complex and abundant microbial communities including bacteria, archaea and fungi [4], [8]–[15]. Recently a great number of investigations have been done to address the functional features of sponge microbes in nitrogen cycle [5], [16]–[21]. Schläppy *et al.* observed nitrification and denitrification in high and low microbial abundance sponges [17]. Diaz and Ward observed that the nitrification capacity of tropical sponges was related to the association between bacteria and sponges [7]. To date, many achievements in nitrification mediated by sponge microbial symbionts have been made, for example ammonia-oxidizing archaea (AOA) [17], [18]–[20], ammonia-oxidizing *Gamma*- and *Betaproteobacteria*, nitrite-oxidizing *Nitrospira* and anaerobic ammonia-oxidizing bacteria *Planctomycetales* [16], [18].

Denitrification is a dissimilatory process in which nitrate and nitrite are reduced to gaseous nitric oxide, nitrous oxide and molecular nitrogen when oxygen is limited, which consists of four reaction steps catalyzed by nitrate reductase (*napA* or *narG*), nitrite reductase (*nirK* or *nirS*), nitric oxide reductase (*qnorB* or *cnorB*) and nitrous oxide reductase (*nosZ*). Nitrite reductase is the key enzyme of this respiratory process since it catalyzes the reduction of soluble nitrite into gas NO. Nitrous oxide reductase catalyses the last step in the complete denitrification pathway. Greenhouse gas N_2_O, which contribute not only to global warming but also directly to the destruction of the stratospheric ozone layer, will go to the atmosphere without the further reduction by nitrous oxide reductase. Therefore nitrite reductase (cytochrome cd1-dependent *nirK* or copper-containing *nirS*) genes and nitrous oxide reductase (*nosZ*) gene are usually used as genetic markers to investigate denitrifying community [22]–[26].

Early in 1979, the nitrogen fixation by sponge cyanobacteria was observed [27]. To date, using nitrogen cycle-related functional genes as markers, the possible roles of sponge microbial symbionts in nitrogen transformation have been suggested, *e.g.* nitrogen fixation (*nifH* gene) [28], ammonia oxidization (*amoA* gene)[18]–[20], [29]–[34], and nitrite reduction(*nir* gene) [5], [33], [34], [35].Hoffmann *et al*. [5] and Schläppy *et al.* [17] detected the N_2_ release from the sponges with high microbial abundances (*Geodia barrette*, *Chondrosia reniformi*s) and low microbial abundance (*Dysidea avara*), however no information of *nosZ* gene (nitrous oxide reduction to N_2_) was provided in these reports. In the genome analysis of a member of the *Poribacteria* from sponge *Aplysina aerophoba*, *nosZ* gene was not found [35]. Until recently, Fan *et al*. detected *nosZ* gene in the metagenomes of some sponges [36]. Therefore, more gene-level molecular evidence for N_2_ release by sponges needs to be provided. Meanwhile, at present, quantitative analysis of nitrogen cycle-related genes is relatively lacking. In this study, using two key functional genes as markers, *nirK* gene encoding nitrite reductase and *nosZ* gene encoding nitrous oxide reductase, the potential of sponge microbiota in the release of N_2_ was investigated. Besides the phylogenetic diversity analysis of *nosZ* and *nirK* genes, their relative quantification was analyzed by real-time qPCR for the first time. This study provides the further understanding of sponge bacterial denitrification potential by the qualitative and quantative analyses of *nirK* and *nosZ* genes, extending our knowledge of nitrogen cycling process in sponges.

## Materials and Methods

### Ethics Statement: N/A

This study and the collection of sponges were approved by the ethics committee at School of Life Sciences and biotechnology, Shanghai Jiao Tong University.

No legislation was required for the sampling of sponges around Yongxing island (112°20′E, 16°50′N). The government of China permits the sampling of sponge samples around the Yongxing island in the South China Sea for scientific research, and no specific permissions were required for these locations/activities, the location is not privately-owned or protected in any way, the field studies did not involve endangered or protected species. We collected the sponge samples ourselves.

### Sponge Sampling

Sponges *Iotrochota* sp., *Xestospongia testudinaria*, *Cinachyrella australiensis* and *Cinachyrella* sp., were collected from the Yongxing Island (112° 20′E, 16° 50′N) in the South China Sea by diving at a depth of *ca.*20 m. Sponges *Amphimedon queenslandica* and *Spheciospongia vesparium* were collected from the Linshui port (110°10′E, 18°24′N) in Hainan province by diving at a depth of *ca*. 20 m. For each sponge species, three individual samples were collected. Samples were placed in bags with natural sea water and transported to the laboratory immediately in an ice-cooled box. The microbes from seawater column on the sponge surface and in inner cavity were removed by washing three times with sterile artificial seawater (ASW) (1.1 g CaCl_2_, 10.2 g MgCl_2_·6 H_2_O, 31.6 g NaCl, 0.75 g KCl, 1.0 g Na_2_SO_4_, 2.4 g Tris-HCl, 0.02 g NaHCO_3_, 1 L distilled water, pH 7.6). Then the sponge samples were stored at –20°C before DNA extraction. The sponge samples were identified according to 28S rRNA or 18S rRNA gene with 99% similarity.

### DNA Extraction and PCR Amplification

Three replicates for each sponge species were used for DNA extraction and the DNA from triplicates was pooled for PCR amplification. Samples were rinsed 3 times by ASW and then homogenized in 1 ml of TE Buffer (10 mMTris, 1 mM EDTA, pH 8.0), centrifuged at 10,000×g for 3 min and grinded using a mortar containing 600 µl CTAB lysisbuffer (2% CTAB, 1.4 M NaCl, 100 mMTris, 20 mMEDTA, 1% PVP) at 65°C. The mycelial mixture was transferred into a 1.5 ml eppendorf tube and heated at 65°C for 30 min, extracted twice with an equal volume of phenol/chloroform/isoamyl alcohol (25∶24:1) and washed with chloroform/isoamyl alcohol (24∶1). After centrifugation at 10, 000×g for 5 min, the supernatant was transferred to a new microtube and precipitated by adding equal volume of isopropanol at −20°C for 1 h. Finally, the DNA pellets were collected by centrifugation (12,000×g, 15 min), washed with75% ethanol twice and re-suspended in 40 µl TE Buffer. RNA was removed by an incubation period with 2 µl of RNase A (10 mg/ml, Invitrogen) at 37°C for10 min. The DNA was quantified by spectrophotometry at 260 nm using a Bio-Photometer (Nano Vue plus, USA).

Fragments of the *nirK*(514 bp) and *nosZ* (454 bp) genes were amplified using the primer pairs nirK1F/nirK5R for *nirK* gene [37] and nosZ-F/nosZ1622R for *nosZ* gene [38] in a final volume of 50 µl using KOD FX (TOYOBO) polymerase system. The PCR mixture was composed of 0.2 µM of each primer, 1×PCR buffer (20 mM Tris-HCl, pH 8.8, 10 mM KCl, 10 mM (NH_4_)_2_SO_4_, 2 mM MgSO_4_), 250 µMdNTP, 2.5UKOD FX and 50–100 ng·µl ^−1^template DNA. PCR was carried out as follows: 2 min at 94°C, followed by 35 cycles of denaturation at 94°C for 30 s, annealing at 57°C (nosZ-F/nosZ1622R) or 56°C(nirK1F/nirK5R) for 40 s, and elongation at 72°C for 40 s. Cycling was completed by a final elongation step of 72°C for 10 min. The PCR products were examined on 1.2% (w/v) ethidium bromide-stained agarose gels.

### Gene Library Construction, Sequencing, and Phylogenetic Analysis

The amplified products were recovered and purified using Agarose Gel DNA Purification Kit (Takara, Dalian). Purified PCR products were cloned with the pEASY-Blunt Zero Cloning kit (TransGen) following the manufacturer’s instructions. The positive recombinants were screened on indicator plates with X-Gal, IPTG and ampicillin by color-based recombinant selection. The positive clones were further identified by sequencing using vector primers M13F/R by Shanghai Majorbio Company. Each clone library has at least 200 positive clones showing the right insert.

All DNA sequences were checked online by NCBI BLAST and aligned using software package Clustal W. The diversity was determined by rarefaction analysis using PHYLIP (Version 3.69). Using DOTUR software, sequences of all *nirK* and *nosZ* gene fragments with similarities >97% were considered as one operational taxonomic unit (OTU) [39]. One representative clone was selected from each OTU for further phylogenetic analysis. According to maximum identity and habitat, all of the OTUs’ nearest neighbors were determined by BLAST analysis. All OTUs’ representative sequences, their nearest neighbors and some reference sequences were imported in MEGA (Version 5) to construct unrooted phylogenetic tree using Neighbor-Joining method.

### Primer Design for Real-time qPCR

For the preparation of real-time qPCR standards, all OTUs’ representative sequences were aligned by software package Clustal W. The real-time qPCR primers were designed using software package Primer Premier 5. Two sets of primers for *nirK* gene were designed for OTU 1 and OTU2 (Table 1). While, three sets of primers were designed for 5 OTUs of *nosZ* gene. The specificity of the primers was tested by PCR, agarose (1.2%) gel electrophoresis and UV translumination after ethidium bromide staining.

**Table 1:** Primer specificity and quantification of standard plasmids.

Target gene	Primer	Nucleotide sequence (5′–3′)	Reference	Annealing temperature°C	Expected size (bp)	Quantification of standard plasmids copies/µl	OTU
	nirk1f	GGMATGGTKCCSTGGCA	[37]	55.5	127	4.8E+10	OTU1
***nirK***	nirk127r	CCTGCTCACCGACATAATAGA	This study				
	nirk1f	GGMATGGTKCCSTGGCA	[37]	56	208	3.5E+10	OTU2
	nirk208r	CCGCAACCGTATCTTCGT	This study				
							
	nosZ130f	CRATGGGTGAAACCAAAGA	This study	55.2	145	3.4E+10	OTU1,2
***nosZ***	nosZ275r	ATGGACCACCTTCATTTCG	This study				
	nosZ-F	CGYTGTTCMTCGACAGCCAG	[38]	57	156	3.9E+10	OTU3
	nosZ156r	CGTCCGCCTCATTGGTCTC	This study				
	nosZ-F	CGYTGTTCMTCGACAGCCAG	[38]	56	201	2.8E+10	OTU4,5
	nosZ201r	GGWAYCGGTCCTTGGAGAAT	This study				

### Standard Curve and Real-time qPCR Assay

The plasmids containing *nirK* and *nosZ* gene fragments were used as standards, which were transformed from Trans1-T1 phage resistant chemically competent cell with specific primers in Table 1. The recombinant plasmids were inoculated into LB broth with ampicillin (100 mg liter^−^1) and incubated at 37°C overnight. Plasmid DNA was then extracted using the Mini BEST Plasmid Purification Kit (Ver.3.0, TaKaRa, Dalian) according to the Manufacturer’s instruction and the plasmid concentrations were determined by spectrophotometry using a BioPhotometer (Nano Vue plus, USA). Standards were prepared from plasmid serial dilutions containing between 10^1^ to 10^9^ copies calculated directly from the concentration of extracted plasmid.

The real-time qPCR was performed in 8-Strip Low Profile tubes (TLS-0851; MJ Research, Watertown MA) and covered with Ultra Clear caps (TCS-0803; MJ Research) on the Real Plex 4S (Eppendorf, Germany). The 25 µl reaction mixture contained SYBR green PCR Master Mix (SYBR Premix EXTaqTM kit, TaKaRa, Dalian), 0.2 µM of primer, 12.5 µl of SYBR Premix EXTaqTM (TaKaRa, Dalian) and 1 µl of template DNA (at 50–100 ng µl ^−1^). Thermal cycling conditions for *nirK* gene were as follows: an initial cycle of 95°C for 30 s; 40 cycles of 95°C for 5 s, 56°Cfor 30 s. The thermal cycling conditions for the *nosZ* primers were similar except for the annealing temperature at 57°C. Thermal cycling, fluorescent data collection and data analysis were carried out with monitor software detection system according to the manufacturer’s instructions. The real-time qPCR assay of the *nosZ* and *nirK* genes were performed independently as described above for different OUT in triplicates (Table 1), the corresponding standard curve was given by specific plasmid. 1 µl of ddH_2_O instead of template DNA was used as negative control.

### Nucleotide Sequence Accession Number

All representative sequences were deposited in GenBank under the accession numbers: JQ823133 and JQ823134 for *nirK* genes, JQ823135, JQ823136, JQ823138, JQ823139 and JQ965748 for *nosZ* genes. The 28S rRNA and 18S rRNA genes of sponge samples were also deposited in GenBank under the accession numbers: KC762728, KC762706, KC762714, KC763778, KC762736, KC774024.

## Results

### Phylogenetical Diversity of Bacterial *nirK* and *nosZ* Genes in Sponges

Two *nirK* and *nosZ* gene clone libraries were successfully constructed. As a result, based on the 23 and 34 clones sequenced, 2 and 5 OTUs were obtained for *nirK* and *nosZ* genes, respectively, at 97% identity level (Table 2; Figure S1).The mean estimations of OTU richness using Chao estimators were 2 and 5.5 OTUs for *nirK* and *nosZ* genes, respectively. Meanwhile, the analysis in Table S1 also suggested the sequencing is nearly saturated, for example the coverage of *nirK* and *nosZ* reached 95.7%, 94.1%, respectively. All the information indicated that sequencing more clones could not greatly increase the actinobacterial diversity because of the conservatism of *nirK* and *nosZ* genes.

**Table 2:** Distribution of clone numbers in *nirK* and *nosZ* gene libraries.

Gene	Family	Reference	Accession number	Similarity %	OTU	Sponge*							Total clone	
						1	2	3	4	5	6			
***nirK***	*Alphaproteobacteria*	Uncultured bacterium from contaminated aquifer	ABP33197	100	OTU1	4	9	1	/	3	5	22		
	*Bataproteobacteria*	*Pusillimonas* sp. T7–7 fromBohai Sea	YP004418446	88	OTU2	/	1	/	/	/	/	1		
***nosZ***	*Alphaproteobacteria*	Uncultured bacterium frombioreactor treatingpiggery waste	ACH69029	99	OTU1	/	1	/	1	/	/	2		
				83	OTU2	3	17	/	2	/	1	23		
		Uncultured bacterium from coastalmarine sediment to Arable Land	ACJ02250	81	OTU3	/	/	/	/	7	/	7		
		Uncultured bacterium frommesotidal sediment in France:Arcachon Bay	CBI71158	88	OTU4	/	/	/	1	/	/	1		
	*Gammaproteobacteria*	Uncultured bacterium from permafrost affected peat soil in arctic tundra	CCA94823	96	OTU5	/	/	/	/	/	1	1		

Note: *Sponge: 1 is *A. queenslandica* from the Linshui port; 2 is *S. vesparium* from the Linshui port; 3 is *Iotrochota* sp. from Yongxing Island; 4 is *X. testudinaria* from Yongxing Island; 5 is *C. australiensis* sp. from Yongxing Island; 6 is *Cinachyrella* sp. from Yongxing Island. The BLAST analysis was based on amino acid sequences.

As shown in Table 2, OTU1 of *nirK* gene was predominated in the gene library with 22 sequences (95.6% of the clones sequenced) from 5 species of sponges except for *X. testudinaria*, while the OTU2 of *nirK* gene was found only one in sponge *S. avesparium*, and particularly, *nirK* gene was not detected in *X. testudinaria*. For *nosZ* genes, *A. queenslandica* and *S. vesparium* had OTU1 and OTU2, *X. testudinaria* had OTU1, 2 and OTU4, *Cinachyrella* sp. had OTU2 and OTU5, whereas OTU3 was observed in *C. australiensis*. In particular, *nosZ* gene was not detected in *Iotrochota* sp. The OTU2 of *nosZ* gene was dominated in the gene library with a highest ratio of 67.6% and it was detected in four species of sponges, *A. queenslandica*, *S. vesparium*, *X. testudinaria* and *Cinachyrella* sp.

**Figure 1:**
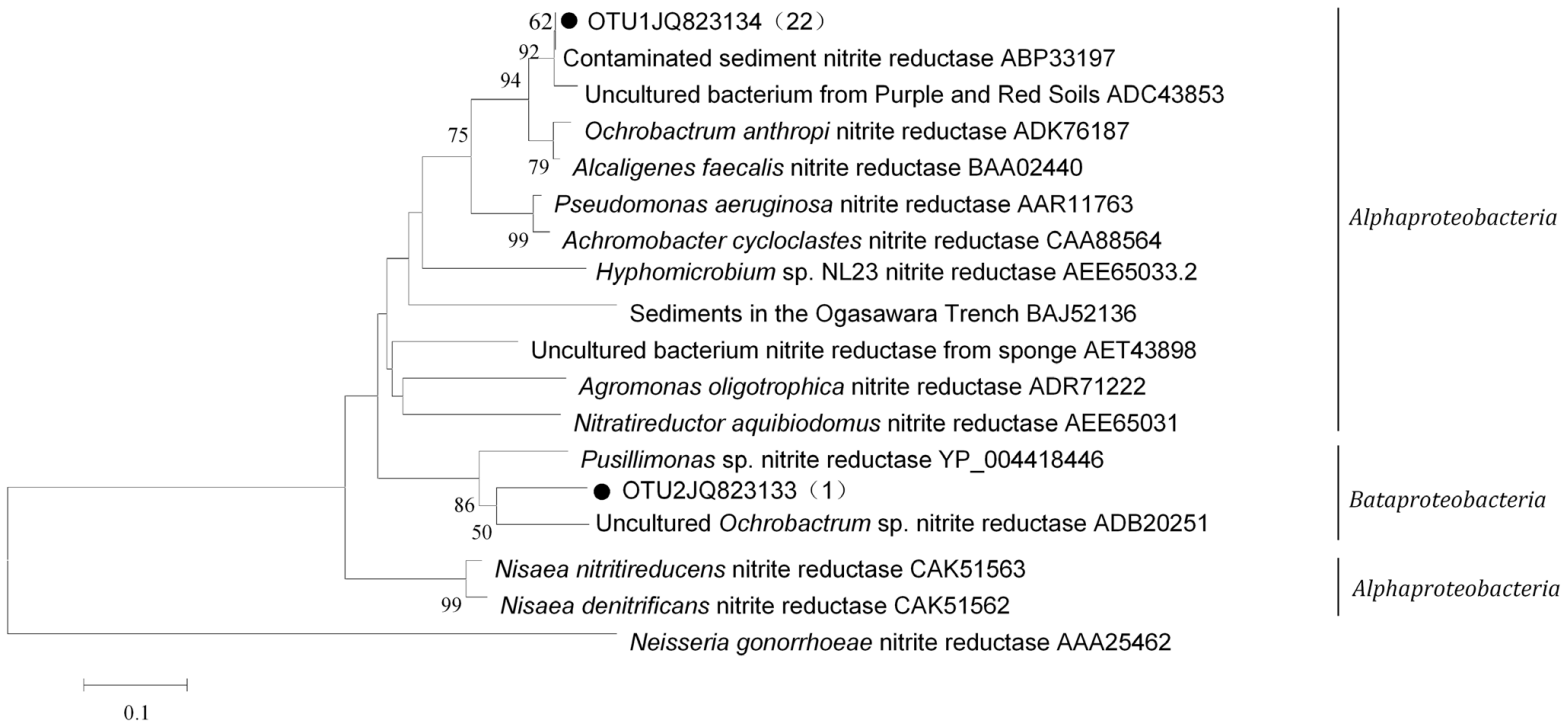
Phylogenetic tree based on amino acid sequence (171 aa) translated from partial gene fragment of *nirK*. The tree is reconstructed using the neighbor-joining method and bootstrap analysis is carried out with 1,000 replicates. Bootstrap values <50% are hidden. The scale bar represents 0.1 AA substitutions per site. The number in parentheses shows the number of sequences in each OTU. •means sequences obtained in this study.

**Figure 2:**
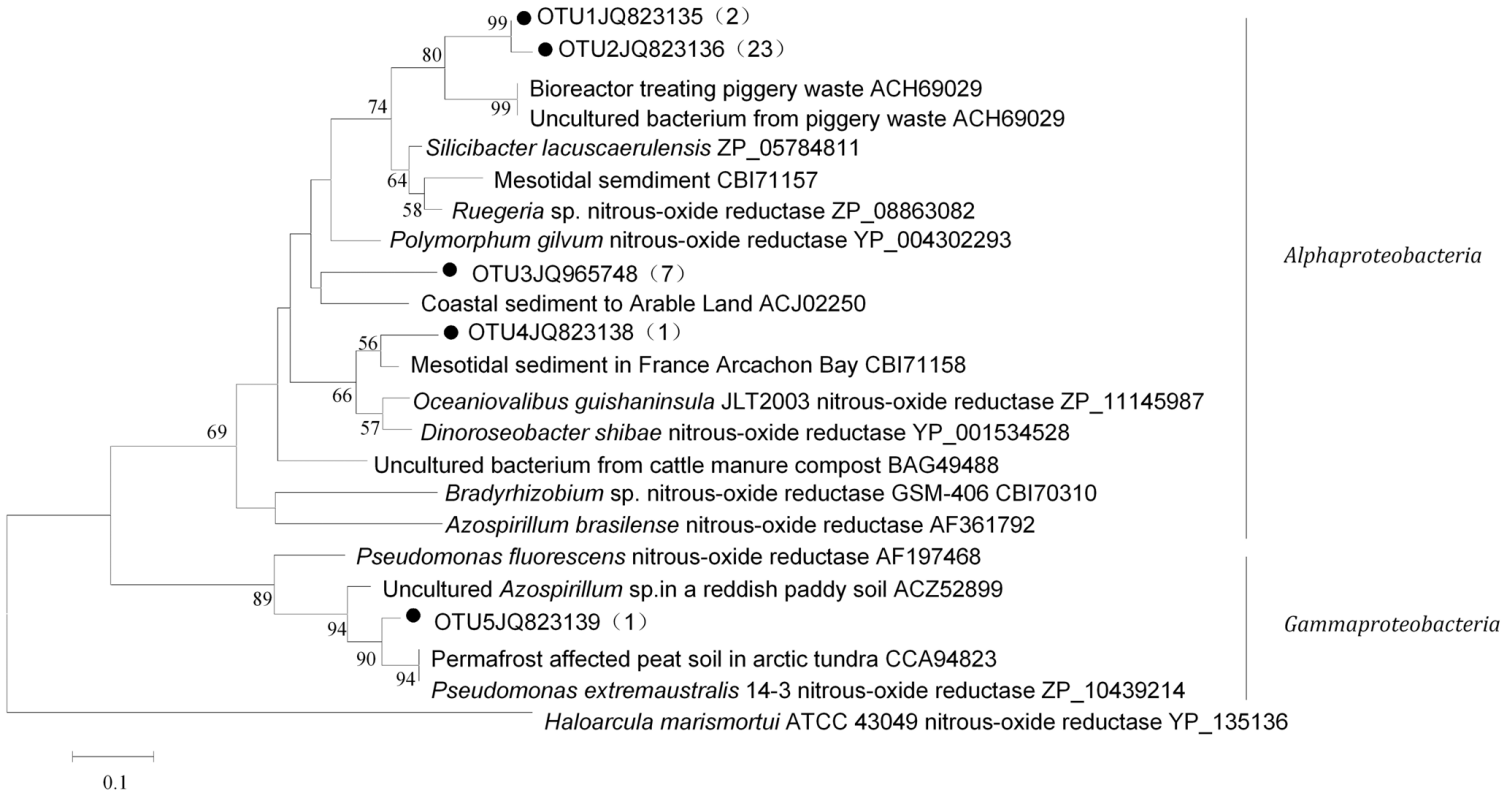
Phylogenetic tree based on amino acid sequence (151 aa) translated from partial gene fragment of *nosZ*. The tree is reconstructed using the neighbor-joining method and bootstrap analysis is carried out with 1,000 replicates. Bootstrap values <50% are hidden. The scale bar represents 0.1 AA substitutions per site. The number in parentheses shows the number of sequences in each OTU. •means sequences obtained in this study.

BLAST and phylogenetic analyses indicated that all the detected *nirK* and *nosZ* genes were probably of *Proteobacteria* origin (Fig. 1, 2).The *nirK* OTU1(95.6% of the clones sequenced) was closely related to *nirK* of an uncultured bacterium with in *Alphaproteobacteria* group in an acidic nitrate and uranium contaminated aquifer in Oak Ridge of USA [40]. Meanwhile, *nirK* OTU1 was closely related to *nirK* of *Ochrobactrum anthropic* isolated from a Paddy Soil [41]. The *nirK* OTU2 contained only one sequence, which belonged to *Betaproteobacteria*. OTU2 was closely related to *nirK* of *Pusillimonas* sp. T7–7 isolated from the Bohai Sea in China [42].

The *nosZ* OTU1 and OTU2 were grouped together with an uncultured bacterium in a bioreactor treating piggery waste [43]. All *nosZ* gene sequences from sponge *C. australiensis* formed an independent group of OTU3, which had 80% similarity with *Polymorphum gilvum* isolated from oil-polluted saline soil [44]. *NosZ* OTU4 was detected only one from *X. testudinaria*. All the above four *nosZ* OTUs were showed closest similarity with the nitrous oxide reductase of *Alphaproteobacteria* (Fig. 2). Only one sequence from sponge *Cinachyrella* sp. formed OTU5, which showed 96% similarity with the nitrous oxide reductase from an uncultured bacterium of *Gammaproteobacteria* group in permafrost affected peat soil in arctic tundra [45].

### Quantification Analysis of *nirK* and *nosZ* Genes in Different Sponges by Real-time qPCR

The abundance of the *nirK* and *nosZ* genes in the sponge microbial symbionts was evaluated by real-time qPCR using total DNA as the template. The standard curve, which was constructed with 10-fold serial dilutions of plasmid, ranged widely from 10^1^ to 10^8^ copies according to different samples. Also, a strong linear relationship between the C_t_ and the log of the starting copy number was demonstrated (R^2^≥0.998). The efficiency of the reaction was obtained between 0.98 and 1.07(supplementary material, Figure S2). Data standard deviation was analyzed (supplementary material, Table S2). All gene copy data acquired from qPCR were then calculated from per µg DNA to per µg dry sponge tissue. As shown in Table 3, different *nirK* gene and *nosZ* gene copies were observed in different sponges. Particularly, sponge *Cinachyrella* sp. showed the highest copies numbers for *nirK* OTU1 (6.21×10^4^ copies of gene sequence/µg sponge sample). In the case of *nosZ* gene, *S. vesparium* had the highest abundance (2.71×10^5^ copies of gene sequence/µg sponge tissue).

**Table 3:** Quantification of *nirK* and *nosZ* genes by qRT-PCR.

Sponge	OTU1 of *nirK* gene copies/µg	OTU2 of *nirK* gene copies/µg	OTU1,2 of *nosZ* gene copies/µg	OTU3 of *nosZ* gene copies/µg	OTU4,5 of *nosZ* gene copies/µg
*A. queenslandica*	1630.2	/	27.8	/	/
*S. vesparium*	1246.1	278.4	2.71E+05	/	/
*Iotrochota* sp.	1.12E+04	/	/	/	/
*X. testudinaria*	/	/	393.1	/	610
*C. australiensis*	5.35E+04	/	/	1.44E+04	/
*Cinachyrella* sp.	6.21E+04	/	1.15E+04	/	1584.1

Note: “/”means no gene copies were detected. The data were gene copies/µg sponge tissue.

**Figure 3:**
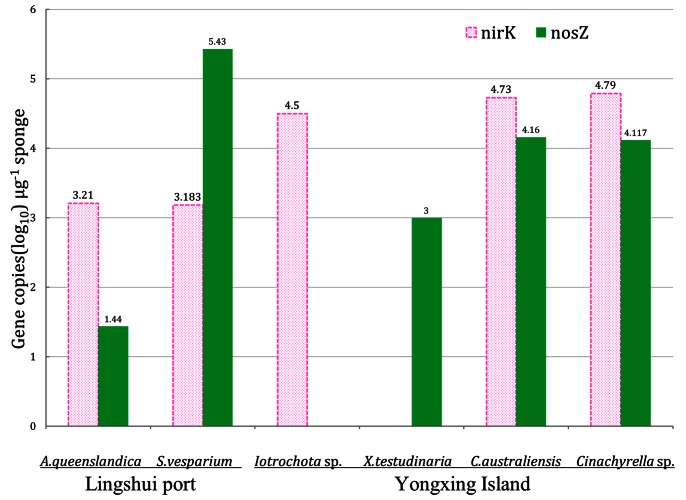
The *nirK* and *nosZ* gene copies in sponges. Sponge names are shown in abscissa axis ordinate shows gene copies treated with log_10_ for per microgramme sponge. The *nirK* gene (OTU1 and OTU2 together) is shown in pink column, while *nosZ* gene (OTU1, OTU2, OTU3, OTU4 and OTU5 together) is shown in green column.

The total abundance comparison of *nirK* and *nosZ* genes is shown in Fig. 3. *A.queenslandica* from Linshui port had a much higher number of *nirK* gene copies than *nosZ* gene. Similarly, *S. vesparium* from the same site showed similar amount of *nirK* gene copies but higher *nosZ* gene copies. *C. australiensis* and *Cinachyrella* sp. from Yongxing Island showed similar abundance of *nirK* and *nosZ* gene copies. The other two sponges from Yongxing Island showed difference in the abundance of two genes: *Iotrochota* sp. showed a higher *nirK* gene copies with the absence of *nosZ* gene copies, whereas, *X. Testudinaria* had a higher *nosZ* gene copies with the absence of *nirK* gene.

## Discussion

### The Phylogenetic Diversity and Abundance of *nirK* Gene and *nosZ* Genes in Sponges

Phylogenetic analysis revealed that most of the *nirK* genes were probably of *Alphaproteobacteria* origin. Only one sequence belonged to *Betaproteobacteria* (Fig. 1). These results agree with Heylen *et al.* who indicated that *nirK* genes were prevalent in *Alphaproteobacteria* [46]. Unlike *nirK* genes, *nosZ* genes were distinctively distributed in different sponges. According to this study, none sponges have sequences in all *nosZ* OTUs and none *nosZ* OTU exists in all sponges. Some sponges only have unique OTU, such as *C. australiensis* has sequences belonging to OTU3 and this OTU does not appearing in other sponges.

Real-time qPCR, is not limited by the cultivability of bacteria and has been successfully applied for the quantification and identification of enteroviruses from surface waters and sponge tissue from the Florida Keys [47]. It was also applied to quantify the relative abundance of unculturable bacterial members from a marine sponge *Vetulina* [48], and to assess *Pseudoalteromonas* species in other marine samples [49]. The abundance of denitrifying bacteria was assessed by quantifying functional genes such as *nirK* and *nosZ* genes from grassland [50], [51], agricultural soil [24], [52], forest [53], glacier foreland [54], riparian [55], arctic soils [45], ocean shore transect [56],and seawater [57]. Using real-time qPCR, among the tested six sponges from two different sea areas, each sponge was found to possess specific abundance of *nirK* and *nosZ* genes.

### The Potential of Sponge Bacterial Symbionts in Marine Nitrogen Balance

In 1979, the potential to fix nitrogen by symbiotic cyanobacteria was demonstrated in Red Sea sponges with the acetylene reduction test [58]. Wilkinson *et al.*demonstrated nitrogen fixation in the Indo-Pacific coral reef sponge *Callyspongia muricina* by the incorporation of ^15^N_2_ into the amino acids of glutamine, glutamate and aspartate [59], [60]. In 2008, Mohamed *et al.* proved the nitrogen fixation by sponge bacterial symbionts based on the expression analysis of nitrogenase gene *nifH* [28]. Therefore, nitrogen fixation by sponge symbionts is possibly an important source of new nitrogen to the reef environment.

Denitrification, as a reverse process of nitrogen fixation and nitrification plays an important role in the balance of marine nitrogen. According to Schläppy *et al*. [17], sponges can change the internal environment such as hypoxia/anoxia or normoxia by water pumping. When sponges stop pumping, their tissues become hypoxic/anoxic and create the microenvironment for denitrification. In general, in the surface water of the oceans, nitrate concentration is very low and nitrite probably comes from the nitrification *e.g.* ammonia oxidization [61], [62]. The surface waters of the oceans are slightly supersaturated with N_2_O [63], because some nitrite is reduced to N_2_O as the final product. Nitrite and N_2_O in sponge body might come from the nitrification and denitrification of sponge microbial symbionts. Inside the oxygen minimum zone in sponge body, nitrite could be reduced to NO and N_2_O and further to N_2_ by denitrifying bacteria with *nirK* or *nosZ* genes, preventing nitrite and N_2_O from accumulating in sponge body. Based on this study, *A. queenslandica*, *S. vesparium*, *C. australiensis*, *Cinachyrella* sp. and *X. testudinaria* could remove the greenhouse gas N_2_O by denitrifying bacteria with *nosZ* genes, which is accompanied by the release of the final product N_2_. The results from this study, together with that from Hofmann *et al*. [5], Schläppy*et al*. [17] and Fan *et al*. [36], indicate the bacterial symbionts of some sponges have similar potential in N_2_ production by denitrification. On the other hand, Fan *et al*. found the denitrification process was not complete for some sponges because *nosZ* gene was lack [36], Similarly, in this study, *nosZ* was not detected in sponge *Lotrochota* sp. So, if there are alternative pathways of nitrous oxide reduction needs further investigation. In addition, this study also suggests the denitrification potential of bacteria varies among sponges because of the different phylogenetic diversity and relative abundance of *nosZ* and *nirK* genes. Ribes *et al*. [64] suggested that unique metabolic pathways were mediated in each sponge species by a different, and host specific, microbial community. The observed different diversity and abundance of *nosZ* and *nirK* genes in this study might come from the different sponge-specific microbes. However, because we did not compare the *nosZ* and *nirK* genes among different samples of one sponge species, so the difference of *nosZ* and *nirK* genes among different individuals of the same sponge species could not be revealed.

Phylogenetic diversity and quantification of denitrification genes are important for a better understanding of denitrifying activity of sponge microbial symbionts in the marine environment. Based on the the qualitative and quantitative analyses of two key functional genes in the complete denitrification process, *nosZ* and *nirK* genes, the different potential of sponge bacterial symbionts in nitrogen gas release was suggested. To our knowledge, this is the first qRT-PCR approach enabling a rapid quantification of functional genes of the denitrifiers in sponges. The limitation of this study lied in the DNA-based approach, only the potential of sponge bacterial symbionts was suggested. The indepth investigation, at RNA level or combined with nitrogen gas detection,will give more details about the ecological roles of sponge bacterial symnbionts in nitrogen gas release.

## Supporting Information

Figure S1 **Rarefaction curves for** ***nirK*** **and** ***nosZ*** **gene sequences.** OTUs are defined at distances of 0.03.
(DOC)

Figure S2 **Quantification of** ***nirK*** **and** ***nosZ*** **genes by Real time qPCR.** Standard curve was constructed with plasmid containing *nirK* or *nosZ* gene sequence for respective OTUs. A: Quantification PCR for OTU1 of *nirK* gene, 5 species of sponges were measured. R^2^ = 0.999 and E = 0.98; B: Quantification PCR for OTU2 of *nirK* gene, 1 species of sponge was measured. R^2^ = 0.999 and E = 0.99; C: Quantification PCR for OTU1 and OTU2 of *nosZ* gene, 4 species of sponges were measured. R^2^ = 0.998 and E = 1.07; D: Quantification PCR for OTU3 of *nosZ* gene, 1 species of sponge was measured. R^2^ = 0.999 and E = 1.03; E: Quantification PCR for OTU4 and OTU5 of *nosZ* gene, 2 species of sponges were measured. R^2^ = 0.998 and E = 1.00.
(DOC)

Table S1 **Phylogenetic information of** ***nirK*** **and** ***nosZ*** **genes libraries.**
(DOC)

Table S2 **Real time qPCR.** A: Real time qPCR of *nirK* gene for OTU1; B: Real time qPCR of *nirK* gene for OTU2; C: Real time qPCR of *nosZ* gene for OTU1 and OTU2; D: Real time qPCR of *nosZ* gene for OTU3; E: Real time qPCR of *nirK* gene for OTU4 and OTU5.
(DOC)

## References

[1] Zehr JP, Kudela RM (2011) Nitrogen cycle of the open ocean: from genes to ecosystems. Ann Rev Mar Sci 3: 197–225. 10.1146/annurev-marine-120709-142819 21329204

[2] Li CW, Chen JY, Hua TE (1998) Precambrian sponges with cellular structures. Science 279: 879–882. 9452391 10.1126/science.279.5352.879

[3] Müller WE, Blumbach B, Müller IM (1999) Evolution of the innate and adaptive immune systems: relationships between potential immune molecules in the lowest metazoan phylum (Porifera) and those invertebrates. Transplantation 68: 1215–1227. 10573054 10.1097/00007890-199911150-00001

[4] Hentschel U, Usher KM, Taylor MW (2006) Marine sponges as microbial fermenters. FEMS Microbiol Ecol 55: 167–177. 16420625 10.1111/j.1574-6941.2005.00046.x

[5] Hoffmann F, Radax R, Woebken D, Holtappels M, Lavik G, et al (2009) Complex nitrogen cycling in the sponge *Geodia barretti* . Environ Microbiol 11: 2228–2243. 19453700 10.1111/j.1462-2920.2009.01944.x

[6] Southwell MW, Popp BN, Martens CS (2008) Nitrification controls on fluxes and isotopic composition of nitrate from Florida Keys sponges. Mar Chem 108: 96–108.

[7] Diaz MC, Ward BB (1997) Sponge-mediated nitrifacation in tropical benthic communities. Mar Ecol Pro Ser 156: 97–107.

[8] Holmes B, Blanch H (2007) Genus-specific associations of marine sponges with group I crenarchaeota. MarBiol 150: 759–772.

[9] Taylor MW, Radax R, Steger D, Wagner M (2007) Sponge-associated microorganisms: evolution, ecology, and biotechnological potential. Microbiol Mol Biol Rev 71: 295–347. 17554047 10.1128/MMBR.00040-06 PMC1899876

[10] Taylor MW, Hill RT, Piel J, Thacker RW, Hentschel U (2007) Soaking it up: the complex lives of marine sponges and their microbial associates. ISME J 1: 187–190. 18043629 10.1038/ismej.2007.32

[11] Lee OO, Lai PY, Wu HX, Zhou XJ, Miao L, et al (2012) *Marinobacter xestospongiae* sp. nov., isolated from the marine sponge *Xestospongia testudinaria* collected from the Red Sea. Int J Syst Evol Microbiol 62: 1980–1985. 22003037 10.1099/ijs.0.028811-0

[12] Schmitt S, Tsai P, Bell J, Fromont J, Ilan M, et al (2012) Assessing the complex sponge microbiota: core, variable and species-specific bacterial communities in marine sponges. ISME J 6: 564–576. 21993395 10.1038/ismej.2011.116 PMC3280146

[13] Taylor MW, Tsai P, Simister RL, Deines P, Botte E, et al (2013) ‘Sponge-specific’ bacteria are widespread (but rare) in diverse marine environments. ISME J 7: 438–443. 23038173 10.1038/ismej.2012.111 PMC3554410

[14] Webster NS, Taylor MW (2012) Marine sponges and their microbial symbionts: love and other relationships. Environ Microbiol 14: 335–346. 21443739 10.1111/j.1462-2920.2011.02460.x

[15] Zhou K, Zhang X, Zhang F, Li Z (2011) Phylogenetically diverse cultivable fungal community and polyketide synthase (PKS), non-ribosomal peptide synthase (NRPS) genes associated with the South China Sea sponges. Microb Ecol 62: 644–654. 21519913 10.1007/s00248-011-9859-y

[16] Mohamed NM, Saito K, Tal Y, Hill RT (2010) Diversity of aerobic and anaerobic ammonia-oxidizing bacteria in marine sponges. ISME J 4: 38–48. 19617876 10.1038/ismej.2009.84

[17] Schläppy ML, Schöttner SI, Lavik G, Kuypers MMM, Beer D, et al (2010) Evidence of nitrification and denitrification in high and low microbial abundance sponges. Mar Bio 157: 593–602. 24391241 10.1007/s00227-009-1344-5 PMC3873014

[18] Bayer K, Schmitt S, Hentschel U (2008) Physiology, phylogeny and in situ evidence for bacterial and archaeal nitrifiers in the marine sponge *Aplysina aerophoba* . Environ Microbiol 10: 2942–2955. 18363713 10.1111/j.1462-2920.2008.01582.x

[19] López-Legentil S, Erwin PM, Pawlik JR, Song B (2010) Effects of sponge bleaching on ammonia-oxidizing archaea: distribution and relative expression of ammonia monooxygenase genes associated with the barrel sponge *Xestospongia muta.*Microb Ecol. 60: 561–571. 10.1007/s00248-010-9662-1 20390264

[20] Steger D, Ettinger-Epstein P, Whalan S, Hentschel U, de Nys R, et al (2008) Diversity and mode of transmission of ammonia-oxidizing archaea in marine sponges. Environ Microbiol 10: 1087–1094. 18177367 10.1111/j.1462-2920.2007.01515.x

[21] Meyer B, Kuever J (2008) Phylogenetic diversity and spatial distribution of the microbial community associated with the Caribbean deep-water sponge *Polymastia cf. corticata* by 16S rRNA, *aprA*, and *amoA* gene analysis. Microb Ecol 56: 306–321. 18193317 10.1007/s00248-007-9348-5 PMC2755779

[22] Liu X, Gao C, Zhang A, Jin P, Wang L, et al (2008) The nos gene cluster from gram-positive bacterium *Geobacillus thermodenitrificans* NG80–2 and functional characterization of the recombinant *nos*Z. FEMS Microbiol Lett 289: 46–52. 19054093 10.1111/j.1574-6968.2008.01362.x

[23] Michotey V, Méjean V, Bonini P (2000) Comparison of methods for quantification of cytochrome cd1-denitrifying bacteria in environmental marine samples. Appl Environ Microbiol 66: 1564–1571. 10742243 10.1128/aem.66.4.1564-1571.2000 PMC92024

[24] Zhou Z, Zheng Y, Shen J, Zhang L, He J (2011) Response of denitrification genes *nirS*, *nirK*, and *nosZ* to irrigation water quality in a Chinese agricultural soil. Environ Sci Pollut Res Int 18: 1644–1652. 21626109 10.1007/s11356-011-0482-8

[25] Braker G, Zhou J, Wu L, Devol AH, Tiedje JM (2000) Nitrite reductase genes (*nirK* and *nirS*) as functional markers to investigate diversity of denitrifying bacteria in pacific northwest marine sediment communities. Appl Environ Microbiol 66: 2096–2104. 10788387 10.1128/aem.66.5.2096-2104.2000 PMC101460

[26] Stres B, Mahne I, Avgustin G, Tiedje JM (2004) Nitrous oxide reductase (*nosZ*) gene fragments differ between native and cultivated Michigan soils. Appl Environ Microbiol 70: 301–309. 14711656 10.1128/AEM.70.1.301-309.2004 PMC321260

[27] Wilkinson CR, Fay P (1979) Nitrogen fixation in coral reef sponges with symbiotic cyanobacteria. Nature 279: 527–529.

[28] Mohamed NM, Colman AS, Tal Y, Hill RT (2008) Diversity and expression of nitrogen fixation genes in bacterial symbionts of marine sponges. Environ Microbiol 10: 2910–2921. 18761667 10.1111/j.1462-2920.2008.01704.x

[29] Radax R, Hoffmann F, Rapp HT, Leininger S, Schleper C (2012) Ammonia-oxidizing archaea as main drivers of nitrification in cold-water sponges. Environ Microbiol 14: 909–923. 22176665 10.1111/j.1462-2920.2011.02661.x

[30] Liu F, Hang M, Zhang F, Zhang B, Li Z (2011) Distribution and abundance of archaea in south China Sea sponge *Holoxea* sp. and the presence of ammonia-oxidizing archaea in sponge cells. Evid-based Comp Alt Med 8: 1–5. 10.1155/2011/723696 PMC3160109 21869898

[31] Turque AS, Batista D, Silveira CB, Cardoso AM, Vieira RP, et al (2010) Environmental shaping of sponge associated archaeal communities. PLOS One 5: e15774. 21209889 10.1371/journal.pone.0015774 PMC3012701

[32] Mohamed N, Saito K, Tal Y, Hill RT (2009) Diversity of aerobic and anaerobic ammonia oxidizing bacteria in marine sponges. ISME J 4: 38–48. 19617876 10.1038/ismej.2009.84

[33] Han M, Li Z**,** Zhang F (2013) The ammonia oxidizing and denitrifying prokaryotes associated with sponges from different sea areas. Microbial Ecology. DOI 10.1007/s00248–013–0197–0. 10.1007/s00248-013-0197-0 23435827

[34] Yang Z, Li Z (2012) Spatial distribution of prokaryotic symbionts and ammoxidation, denitrifier bacteria in marine sponge *Astrosclera willeyana* . Sci Rep 2: 528. 22829982 10.1038/srep00528 PMC3402844

[35] Siegl A, Kamke J, Hochmuth T, Piel J, Richter M, et al (2011) Single-cell genomics reveals the lifestyle of *Poribacteria*, a candidate phylum symbioticallyassociated with marine sponges. The ISME J. 5: 61–70. 10.1038/ismej.2010.95 PMC3105677 20613790

[36] Fan L, Reynolds D, Liu M, Stark M, Kjellebery S, et al.. (2012) Functional equivalence and evolutionary convergence in complex communities of microbial sponge symbionts PNAS doi:10.1073/pnas.1203287109. 10.1073/pnas.1203287109 PMC3390844 22699508

[37] Braker G, Fesefeldt A, Witzel KP (1998) Development of PCR primer systems for amplification of nitrite reductase genes (*nirK* and *nirS*) to detect denitrifying bacteria in environmental samples. Appl Environ Microbiol 64: 3769–3775. 9758798 10.1128/aem.64.10.3769-3775.1998 PMC106545

[38] Kloos K, Mergel A, Rösch C, Bothe H (2001) Denitrification within the genus *Azospirillum* and other associative bacteria. J Plant Physiol28: 991–998.

[39] Palmer K, Drake HL, Horn MA (2009) Genome-derived criteria for assigning environmental *narG* and *nosZ* sequences to operational taxonomic units of nitrate reducers. Appl Environ Microbiol 75: 5170–5174. 19502444 10.1128/AEM.00254-09 PMC2725509

[40] Spain AM, Peacock AD, Istok JD, Elshahed MS, Najar FZ, et al (2007) Identification and isolation of a *Castellaniella* species important during biostimulation of an acidic nitrate- and uranium-contaminated aquifer. Appl Environ Microbiol 73: 4892–4904. 17557842 10.1128/AEM.00331-07 PMC1951013

[41] Chen Z, Luo X, Hu R, Wu M, Wu J, et al (2010) Impact of long-term fertilization on the composition of denitrifier communities based on nitrite reductase analyses in a paddy soil. Microb Ecol 60: 850–861. 20563573 10.1007/s00248-010-9700-z

[42] Cao B, Ma T, Ren Y, Ren Y, Li G, et al (2011) Complete genome sequence of *Pusillimonas* sp. T7–7, a cold-tolerant diesel oil-degrading bacterium isolated from the Bohai Sea in China. J Bacteriol 193: 4021–4022. 21622753 10.1128/JB.05242-11 PMC3147516

[43] Anceno AJ, Rouseau P, Beline F, Shipin OV, Dabert P (2009) Evolution of N-converting bacteria during the start-up of anaerobic digestion coupled biological nitrogen removal pilot-scale bioreactors treating high-strength animal waste slurry. Biores Technol 100: 3678–3687. 10.1016/j.biortech.2009.02.045 19329298

[44] Li S, Tang Y, Nie Y, Cai M, Wu X (2011) Complete genome sequence of *Polymorphumgilvum* SL003B-26A1T, a crude oil-degrading bacterium from oil-polluted saline soil. J Bacteriol 193: 2894–2895. 21478361 10.1128/JB.00333-11 PMC3133105

[45] Palmer K, Biasi C, Horn MA (2012) Contrasting denitrifier communities relate to contrasting N_2_O emission patterns from acidic peat soils in arctic tundra. ISME J 6: 1058–1077. 22134649 10.1038/ismej.2011.172 PMC3329112

[46] Heylen K, Gevers D, Vanparys B, Wittebolle L, Geets J, et al (2006) The incidence of *nirS* and *nirK* and their genetic heterogeneity in cultivated denitrifiers. Environ Microbiol 8: 2012–2021. 17014499 10.1111/j.1462-2920.2006.01081.x

[47] Donaldson KA, Griffin DW, Paul JH (2002) Detection, quantitation and identification of enteroviruses from surface waters and sponge tissue from the Florida Keys using real-time RT-PCR. Water Res 36: 2505–2514. 12153016 10.1016/s0043-1354(01)00479-1

[48] Cassler M, Peterson CL, Ledger A, Pomponi SA, Wright AE, et al (2008) Use of real-time qPCR to quantify members of the unculturable heterotrophic bacterial community in a deep sea marine sponge, *Vetulina* sp. Microb Ecol 55: 384–394. 17661179 10.1007/s00248-007-9283-5

[49] Skovhus TL, Ramsing NB, Holmstrom C, Kjelleberg S, Dahllof I (2004) Real-time quantitative PCR for assessment of abundance of *Pseudoalteromonas* species in marine samples. Appl Environ Microbiol 70: 2373–2382. 15066834 10.1128/AEM.70.4.2373-2382.2004 PMC383141

[50] Philippot L, Cuhel J, Saby NP, Cheneby D, Chronakova A, et al (2009) Mapping field-scale spatial patterns of size and activity of the denitrifier community. Environ Microbiol 11: 1518–1526. 19260937 10.1111/j.1462-2920.2009.01879.x

[51] Cuhel J, Simek M, Laughlin RJ, Bru D, Cheneby D, et al (2010) Insights into the effect of soil pH on N_2_O and N_2_ emissions and denitrifier community size and activity. Appl Environ Microbiol 76: 1870–1878. 20118356 10.1128/AEM.02484-09 PMC2838029

[52] Chen Z, Hou H, Zheng Y, Qin H, Zhu Y, et al (2012) Influence of fertilisation regimes on a *nosZ*-containing denitrifying community in a rice paddy soil. J Sci Food Agric 92: 1064–1072. 21796637 10.1002/jsfa.4533

[53] Levy-Booth DJ, Winder RS (2010) Quantification of nitrogen reductase and nitrite reductase genes in soil of thinned and clear-cut Douglas-fir stands by using real-time PCR. Appl Environ Microbiol 76: 7116–7125. 20802070 10.1128/AEM.02188-09 PMC2976274

[54] Kandeler E, Deiglmayr K, Tscherko D, Bru D, Philippot L (2006) Abundance of *narG*, *nirS*, *nirK*, and *nosZ* genes of denitrifying bacteria during primary successions of a glacier foreland. Appl Environ Microbiol 72: 5957–5962. 16957216 10.1128/AEM.00439-06 PMC1563666

[55] Wu L, Osmond DL, Graves AK, Burchell MR, Duckworth OW (2012) Relationships between nitrogen transformation rates and gene abundance in a riparian buffer soil. Environ Manage 50: 861–874. 22996400 10.1007/s00267-012-9929-z

[56] Lund MB, Smith JM, Francis CA (2012) Diversity, abundance and expression of nitrite reductase (*nirK*)-like genes in marine thaumarchaea. ISME J 6: 1966–1977. 22592819 10.1038/ismej.2012.40 PMC3446794

[57] Auclair J, Parent S, Villemur R (2012) Functional diversity in the denitrifying biofilm of the methanol-fed marine denitrification system at the Montreal Biodome. Microb Ecol 63: 726–735. 22006549 10.1007/s00248-011-9960-2

[58] Wilkinson CR (1979) Nutrient translocation from symbiotic cyanobacteria to coral reef sponges. In: Levi C, Boury-Esnault N (eds). Colloques international du CNRS no.291 - Biologie des spongiaires: Centre Nationl de la Recherche Scientifique. Paris. 373–380.

[59] Wilkinson CR (1983) Net primary productivity of coral reef sponges. Science 219: 410–412. 17815320 10.1126/science.219.4583.410

[60] Wilkinson CLO, Cesar H, Hodgson G, Rubens J, Strong AE (1999) Ecological and socioeconomic impacts of 1998 coral mortality in the Indian Ocean: An ENSO impact and a warning of future change? Ambio 28: 188–196.

[61] Gruber N, Galloway JN (2008) An earth-system perspective of the global nitrogen cycle. Nature 451: 293–296. 18202647 10.1038/nature06592

[62] Gruber N (2008) The marine nitrogen cycle: overview and challenges. in: Douglas GC, Deborah AB, Margaret RM, Edward JC (eds). Nitrogen in the Marine Environment (2nd edition): Academic Press 1–50.

[63] Zehr JP, Ward BB (2002) Nitrogen cycling in the ocean: new perspectives on processes and paradigms. Appl Environ Microbiol 68: 1015–1024. 11872445 10.1128/AEM.68.3.1015-1024.2002 PMC123768

[64] Ribes M, Jiménez E, Yahel G, López-Sendino P, Diez B, et al (2012) Functional convergence of microbes associated with temperate marine sponges. Environm Microbiol 14: 1224–1239. 10.1111/j.1462-2920.2012.02701.x 22335606

