# Supplementary material for: The Different Potential of Sponge Bacterial Symbionts in N2 Release Indicated by the Phylogenetic Diversity and Abundance Analyses of Denitrification Genes, nirK and nosZ
Source: PLoS One. 2013 Jun 10;8(6):e65142. doi: 10.1371/journal.pone.0065142 (PMC3677918; doi:10.1371/journal.pone.0065142)
Supplement: Figure S1 — Rarefaction curves for nirK and nosZ gene sequences. OTUs are defined at distances of 0.03. (DOC) [file pone.0065142.s001.doc]

**Supporting Information**

**Figure S1.** Rarefaction curves for *nirK* and *nosZ* gene sequences. OTUs are defined at distances of 0.03.

**
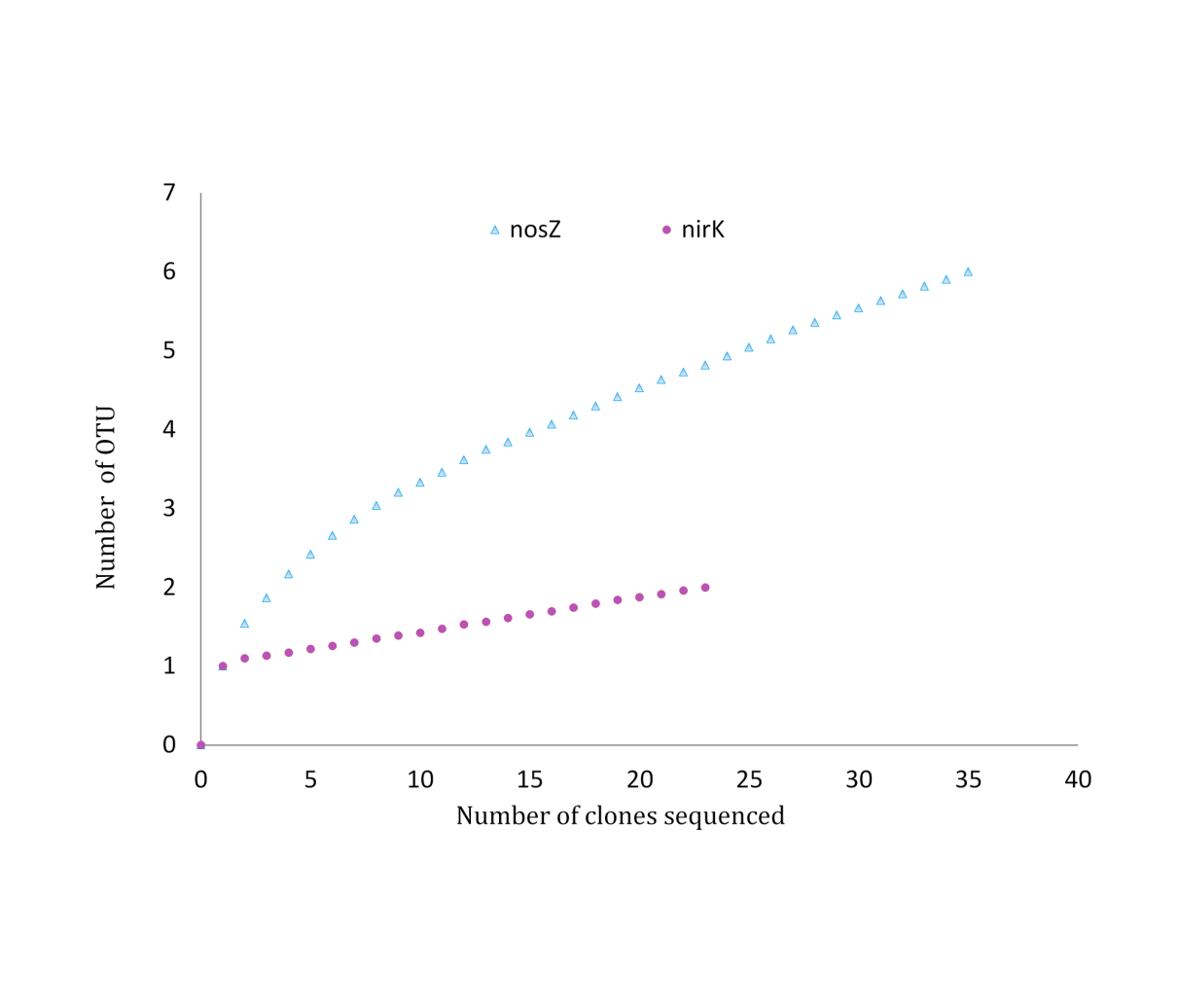
**
