# Supplementary material for: The Different Potential of Sponge Bacterial Symbionts in N2 Release Indicated by the Phylogenetic Diversity and Abundance Analyses of Denitrification Genes, nirK and nosZ
Source: PLoS One. 2013 Jun 10;8(6):e65142. doi: 10.1371/journal.pone.0065142 (PMC3677918; doi:10.1371/journal.pone.0065142)

**Supporting Information**

**Figure S2.** Quantification of *nirK* and *nosZ* genes by Real time qPCR. Standard curve was constructed with plasmid containing *nirK* or *nosZ* gene sequence for respective OTUs. A: Quantification PCR for OTU1 of *nirK* gene, 5 species of sponges were measured. R^2^= 0.999 and E=0.98; B: Quantification PCR for OTU2 of *nirK* gene, 1 species of sponge was measured. R^2^= 0.999 and E=0.99; C: Quantification PCR for OTU1 and OTU2 of *nosZ* gene, 4 species of sponges were measured. R^2^= 0.998 and E=1.07; D: Quantification PCR for OTU3 of *nosZ* gene, 1 species of sponge was measured. R^2^= 0.999 and E=1.03; E: Quantification PCR for OTU4 and OTU5 of *nosZ* gene, 2 species of sponges were measured. R^2^= 0.998 and E=1.00.


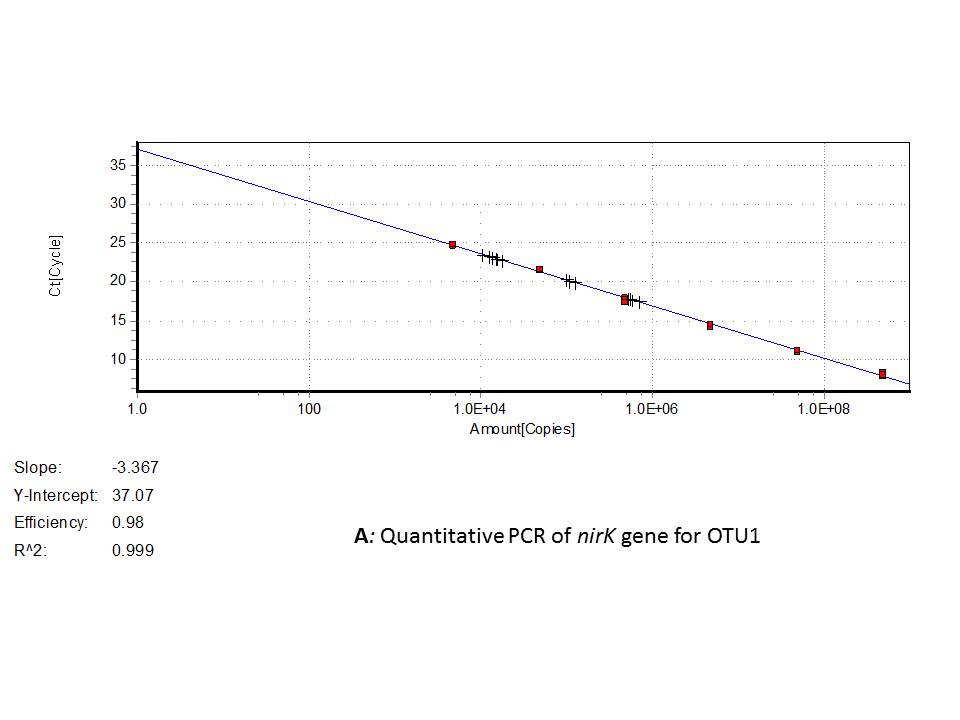


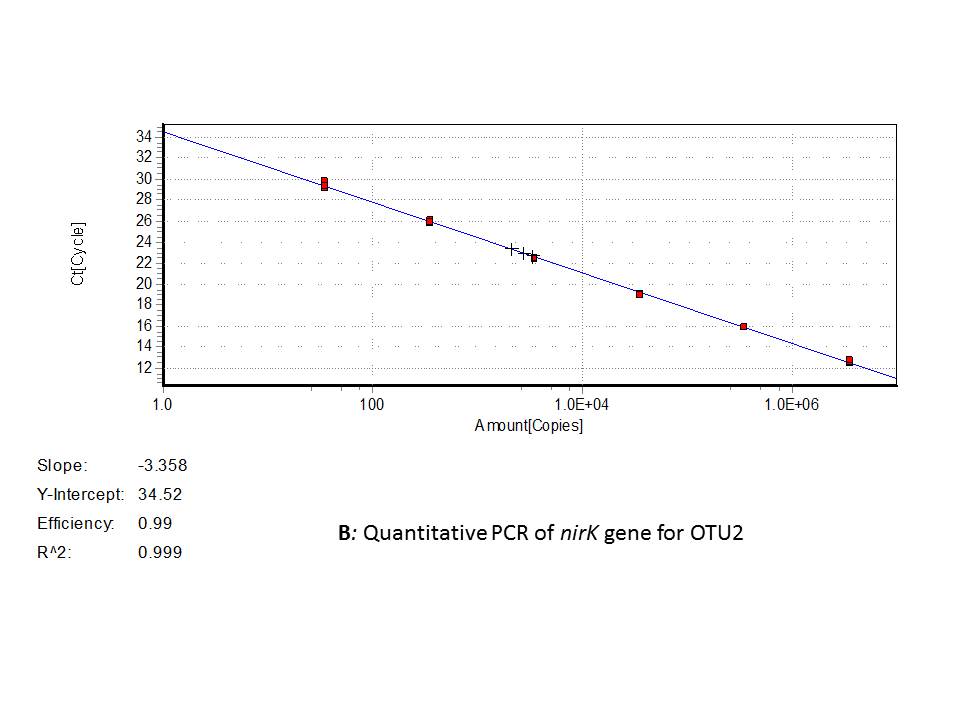


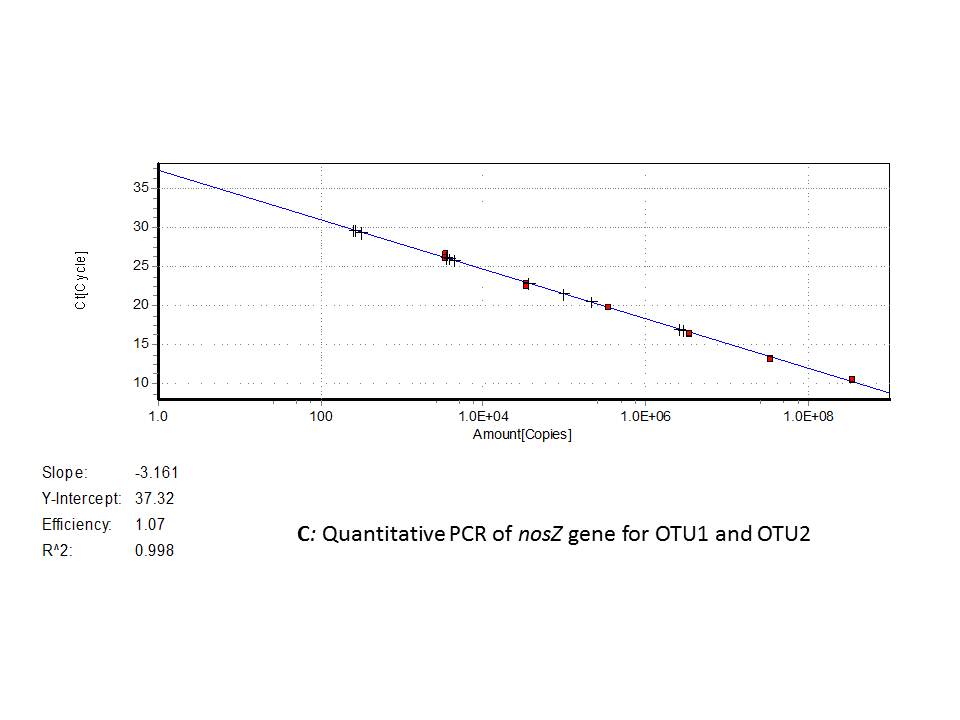


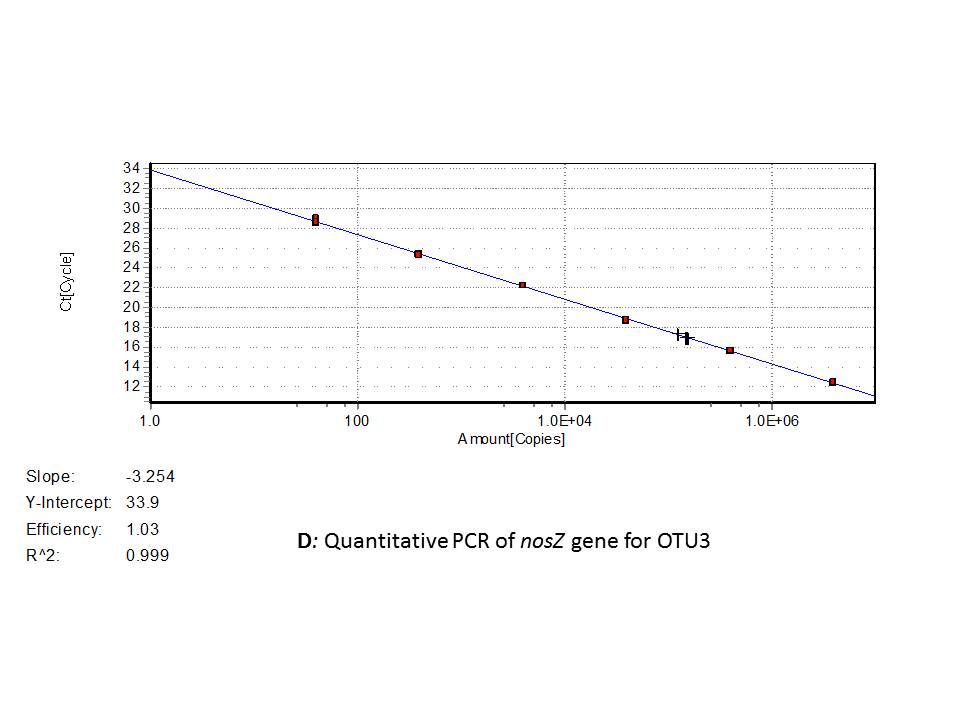


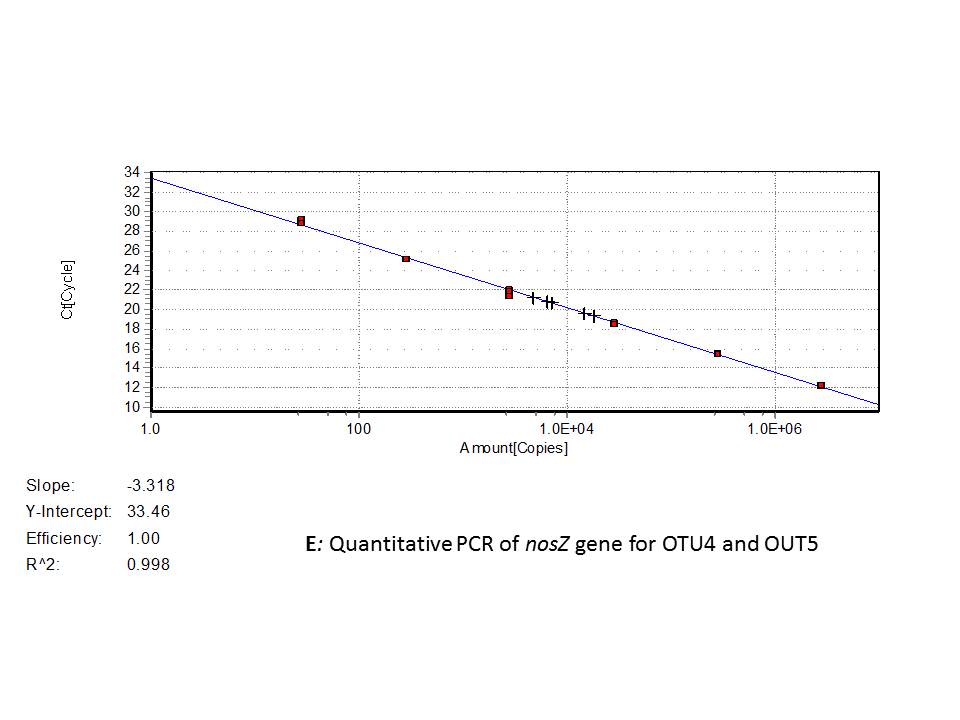

Supplement: Figure S2 — Quantification of nirK and nosZ genes by Real time qPCR. Standard curve was constructed with plasmid containing nirK or nosZ gene sequence for respective OTUs. A: Quantification PCR for OTU1 of nirK gene, 5 species of sponges were measured. R2 = 0.999 and E = 0.98; B: Quantification PCR for OTU2 of nirK gene, 1 species of sponge was measured. R2 = 0.999 and E = 0.99; C: Quantification PCR for OTU1 and OTU2 of nosZ gene, 4 species of sponges were measured. R2 = 0.998 and E = 1.07; D: Quantification PCR for OTU3 of nosZ gene, 1 species of sponge was measured. R2 = 0.999 and E = 1.03; E: Quantification PCR for OTU4 and OTU5 of nosZ gene, 2 species of sponges were measured. R2 = 0.998 and E = 1.00. (DOC) [file pone.0065142.s002.doc]
