# Supplementary material for: The Different Potential of Sponge Bacterial Symbionts in N2 Release Indicated by the Phylogenetic Diversity and Abundance Analyses of Denitrification Genes, nirK and nosZ
Source: PLoS One. 2013 Jun 10;8(6):e65142. doi: 10.1371/journal.pone.0065142 (PMC3677918; doi:10.1371/journal.pone.0065142)
Supplement: Table S1 — Phylogenetic information of nirK and nosZ genes libraries. (DOC) [file pone.0065142.s003.doc]

**Supporting Information**

**Table S1.** Phylogenetic information of *nirK* and *nosZ* genes libraries

| Gene | Read number | Distance^1^ (cutoff) | OTUs (richness^2^) | ACE^3^ | Chao^4^ | Shannon^5^ | 1/Simpson^6^ | Coverage^7^% |
| --- | --- | --- | --- | --- | --- | --- | --- | --- |
| *nirK* | 23 | 0.03 | 2 | 0 | 2.0 | 0.18 | 1.10 | 95.7 |
| *nosZ* | 34 | 0.03 | 5 | 8.22 | 5.5 | 0.96 | 2.04 | 94.1 |

Note: ^1^97% identity was estimated as the species-level distance for *nirK* and *nosZ* gene respectively.

^2^Richness is based on observed unique OTUs.

^3^Nonparametric statistical prediction of total richness of different OTUs based on distribution of abundant (>10) and rare (≤10) OTUs.

^4^Nonparametric statistical prediction of total richness of different OTUs based on distribution of singletons and doubletons.

^5^Shannon diversity index. A higher number represents higher diversity.

^6^1/Simpson’s diversity index. A higher number represents higher diversity.

^7^Percentage of coverage, percentage of observed number of OTUs divided by Chao estimate.
