# Supplementary material for: The Different Potential of Sponge Bacterial Symbionts in N2 Release Indicated by the Phylogenetic Diversity and Abundance Analyses of Denitrification Genes, nirK and nosZ
Source: PLoS One. 2013 Jun 10;8(6):e65142. doi: 10.1371/journal.pone.0065142 (PMC3677918; doi:10.1371/journal.pone.0065142)
Supplement: Table S2 — Real time qPCR. A: Real time qPCR of nirK gene for OTU1; B: Real time qPCR of nirK gene for OTU2; C: Real time qPCR of nosZ gene for OTU1 and OTU2; D: Real time qPCR of nosZ gene for OTU3; E: Real time qPCR of nirK gene for OTU4 and OTU5. (DOC) [file pone.0065142.s004.doc]

**Supporting Information**

**Table S2.** Real time qPCR

**A:** Real time qPCR of *nirK* gene for OTU1

| **Name** | **Ct SYBR** | **Ct Mean SYBR** | **Ct Dev. SYBR** | **Amount SYBR [Copies]** | **Amount Mean SYBR** | **Amount Dev. SYBR** |
| --- | --- | --- | --- | --- | --- | --- |
| **Std_4.8E8/1** | 7.89 | 8.06 | 0.25 | 4.80E+08 |  |  |
| **Std_4.8E8/2** | 8.35 | 8.06 | 0.25 | 4.80E+08 |  |  |
| **Std_4.8E8/3** | 7.93 | 8.06 | 0.25 | 4.80E+08 |  |  |
| **1/1** | 22.94 | 22.9 | 0.1 | 15793 | 16302 | 1136 |
| **1/2** | 22.97 | 22.9 | 0.1 | 15509 | 16302 | 1136 |
| **1/3** | 22.78 | 22.9 | 0.1 | 17603 | 16302 | 1136 |
| **Std_4.8E7/1** | 11.17 | 11.14 | 0.03 | 4.80E+07 |  |  |
| **Std_4.8E7/2** | 11.12 | 11.14 | 0.03 | 4.80E+07 |  |  |
| **Std_4.8E7/3** | 11.13 | 11.14 | 0.03 | 4.80E+07 |  |  |
| **2/1** | 23.24 | 23.29 | 0.2 | 12871 | 12461 | 1631 |
| **2/2** | 23.51 | 23.29 | 0.2 | 10664 | 12461 | 1631 |
| **2/3** | 23.13 | 23.29 | 0.2 | 13848 | 12461 | 1631 |
| **Std_4.8E6/1** | 14.62 | 14.44 | 0.25 | 4.80E+06 |  |  |
| **Std_4.8E6/2** | 14.15 | 14.44 | 0.25 | 4.80E+06 |  |  |
| **Std_4.8E6/3** | 14.53 | 14.44 | 0.25 | 4.80E+06 |  |  |
| **3/1** | 19.89 | 20.08 | 0.18 | 1.27E+05 | 1.12E+05 | 14048 |
| **3/2** | 20.1 | 20.08 | 0.18 | 1.10E+05 | 1.12E+05 | 14048 |
| **3/3** | 20.25 | 20.08 | 0.18 | 99604 | 1.12E+05 | 14048 |
| **Std_480000/1** | 17.91 | 17.72 | 0.21 | 4.80E+05 |  |  |
| **Std_480000/2** | 17.76 | 17.72 | 0.21 | 4.80E+05 |  |  |
| **Std_480000/3** | 17.49 | 17.72 | 0.21 | 4.80E+05 |  |  |
| **5/1** | 17.79 | 17.79 | 0.02 | 5.36E+05 | 5.35E+05 | 6597 |
| **5/2** | 17.77 | 17.79 | 0.02 | 5.41E+05 | 5.35E+05 | 6597 |
| **5/3** | 17.81 | 17.79 | 0.02 | 5.28E+05 | 5.35E+05 | 6597 |
| **Std_48000/1** | 21.42 | 21.48 | 0.09 | 48000 |  |  |
| **Std_48000/2** | 21.44 | 21.48 | 0.09 | 48000 |  |  |
| **Std_48000/3** | 21.58 | 21.48 | 0.09 | 48000 |  |  |
| **6/1** | 17.36 | 17.58 | 0.2 | 7.19E+05 | 6.21E+05 | 88705 |
| **6/2** | 17.63 | 17.58 | 0.2 | 5.97E+05 | 6.21E+05 | 88705 |
| **6/3** | 17.76 | 17.58 | 0.2 | 5.47E+05 | 6.21E+05 | 88705 |
| **Std_4800/1** | 24.61 | 24.76 | 0.14 | 4800 |  |  |
| **Std_4800/2** | 24.77 | 24.76 | 0.14 | 4800 |  |  |
| **Std_4800/3** | 24.89 | 24.76 | 0.14 | 4800 |  |  |
| **n** | / |  |  | / |  |  |

**B:** Real time qPCR of *nirK* gene for OTU2

| **Name** | **Ct SYBR** | **Ct Mean SYBR** | **Ct Dev. SYBR** | **Amount SYBR [Copies]** | **Amount Mean SYBR** | **Amount Dev. SYBR** |
| --- | --- | --- | --- | --- | --- | --- |
| **Std_3.5E6/1** | 12.54 | 12.66 | 0.14 | 3.50E+06 |  |  |
| **Std_3.5E6/2** | 12.64 | 12.66 | 0.14 | 3.50E+06 |  |  |
| **Std_3.5E6/3** | 12.81 | 12.66 | 0.14 | 3.50E+06 |  |  |
| **2/1** | 22.93 | 22.98 | 0.35 | 2819 | 2784 | 654 |
| **2/2** | 22.65 | 22.98 | 0.35 | 3420 | 2784 | 654 |
| **2/3** | 23.35 | 22.98 | 0.35 | 2113 | 2784 | 654 |
| **Std_350000/1** | 15.95 | 15.96 | 0.02 | 3.50E+05 |  |  |
| **Std_350000/2** | 15.94 | 15.96 | 0.02 | 3.50E+05 |  |  |
| **Std_350000/3** | 15.99 | 15.96 | 0.02 | 3.50E+05 |  |  |
| **Std_35000/1** | 19.09 | 19.05 | 0.04 | 35000 |  |  |
| **Std_35000/2** | 19.06 | 19.05 | 0.04 | 35000 |  |  |
| **Std_35000/3** | 19 | 19.05 | 0.04 | 35000 |  |  |
| **Std_3500/1** | 22.59 | 22.51 | 0.09 | 3500 |  |  |
| **Std_3500/2** | 22.42 | 22.51 | 0.09 | 3500 |  |  |
| **Std_3500/3** | 22.5 | 22.51 | 0.09 | 3500 |  |  |
| **Std_350/1** | 26.08 | 25.98 | 0.1 | 350 |  |  |
| **Std_350/2** | 25.89 | 25.98 | 0.1 | 350 |  |  |
| **Std_350/3** | 25.97 | 25.98 | 0.1 | 350 |  |  |
| **Std_35/1** | 29.21 | 29.47 | 0.3 | 35 |  |  |
| **Std_35/2** | 29.79 | 29.47 | 0.3 | 35 |  |  |
| **Std_35/3** | 29.4 | 29.47 | 0.3 | 35 |  |  |
| **n** | / |  |  | / |  |  |

**C:** Real time qPCR of *nosZ* gene for OTU1 and OTU2

| **Name** | **Ct SYBR** | **Ct Mean SYBR** | **Ct Dev. SYBR** | **Amount SYBR [Copies]** | **Amount Mean SYBR** | **Amount Dev. SYBR** |
| --- | --- | --- | --- | --- | --- | --- |
| **Std_3.4E8/1** | 10.64 | 10.66 | 0.06 | 3.40E+08 |  |  |
| **Std_3.4E8/2** | 10.72 | 10.66 | 0.06 | 3.40E+08 |  |  |
| **Std_3.4E8/3** | 10.6 | 10.66 | 0.06 | 3.40E+08 |  |  |
| **1/1** | 29.72 | 29.6 | 0.17 | 253 | 278 | 35.2 |
| **1/2** | 29.67 | 29.6 | 0.17 | 263 | 278 | 35.2 |
| **1/3** | 29.4 | 29.6 | 0.17 | 318 | 278 | 35.2 |
| **Std_3.4E7/1** | 13.3 | 13.28 | 0.05 | 3.40E+07 |  |  |
| **Std_3.4E7/2** | 13.23 | 13.28 | 0.05 | 3.40E+07 |  |  |
| **Std_3.4E7/3** | 13.32 | 13.28 | 0.05 | 3.40E+07 |  |  |
| **2/1** | 16.9 | 16.98 | 0.07 | 2.88E+06 | 2.71E+06 | 1.49E+05 |
| **2/2** | 17.03 | 16.98 | 0.07 | 2.60E+06 | 2.71E+06 | 1.49E+05 |
| **2/3** | 17.02 | 16.98 | 0.07 | 2.64E+06 | 2.71E+06 | 1.49E+05 |
| **Std_3.4E6/1** | 16.58 | 16.51 | 0.09 | 3.40E+06 |  |  |
| **Std_3.4E6/2** | 16.54 | 16.51 | 0.09 | 3.40E+06 |  |  |
| **Std_3.4E6/3** | 16.41 | 16.51 | 0.09 | 3.40E+06 |  |  |
| **4/1** | 25.99 | 25.96 | 0.16 | 3834 | 3931 | 460 |
| **4/2** | 25.79 | 25.96 | 0.16 | 4432 | 3931 | 460 |
| **4/3** | 26.1 | 25.96 | 0.16 | 3527 | 3931 | 460 |
| **Std_3.4E5/1** | 19.89 | 19.87 | 0.03 | 3.40E+05 |  |  |
| **Std_3.4E5/2** | 19.88 | 19.87 | 0.03 | 3.40E+05 |  |  |
| **Std_3.4E5/3** | 19.83 | 19.87 | 0.03 | 3.40E+05 |  |  |
| **6/1** | 22.9 | 21.64 | 1.21 | 36334 | 1.15E+05 | 87717 |
| **6/2** | 20.49 | 21.64 | 1.21 | 2.10E+05 | 1.15E+05 | 87717 |
| **6/3** | 21.53 | 21.64 | 1.21 | 98802 | 1.15E+05 | 87717 |
| **Std_34000/1** | 22.58 | 22.73 | 0.14 | 34000 |  |  |
| **Std_34000/2** | 22.87 | 22.73 | 0.14 | 34000 |  |  |
| **Std_34000/3** | 22.73 | 22.73 | 0.14 | 34000 |  |  |
| **Std_3400/1** | 26.23 | 26.45 | 0.24 | 3400 |  |  |
| **Std_3400/2** | 26.41 | 26.45 | 0.24 | 3400 |  |  |
| **Std_3400/3** | 26.7 | 26.45 | 0.24 | 3400 |  |  |
| **n** | / |  |  | / |  |  |

**D:** Real time qPCR of *nosZ* gene for OTU3

| **Name** | **Ct SYBR** | **Ct Mean SYBR** | **Ct Dev. SYBR** | **Amount SYBR [Copies]** | **Amount Mean SYBR** | **Amount Dev. SYBR** |
| --- | --- | --- | --- | --- | --- | --- |
| **Std_3.9E6/1** | 12.59 | 12.52 | 0.08 | 3.90E+06 |  |  |
| **Std_3.9E6/2** | 12.44 | 12.52 | 0.08 | 3.90E+06 |  |  |
| **Std_3.9E6/3** | 12.53 | 12.52 | 0.08 | 3.90E+06 |  |  |
| **5/1** | 17.04 | 17.12 | 0.17 | 1.51E+05 | 1.44E+05 | 17149 |
| **5/2** | 17.32 | 17.12 | 0.17 | 1.24E+05 | 1.44E+05 | 17149 |
| **5/3** | 17 | 17.12 | 0.17 | 1.56E+05 | 1.44E+05 | 17149 |
| **Std_390000/1** | 15.7 | 15.73 | 0.07 | 3.90E+05 |  |  |
| **Std_390000/2** | 15.68 | 15.73 | 0.07 | 3.90E+05 |  |  |
| **Std_390000/3** | 15.81 | 15.73 | 0.07 | 3.90E+05 |  |  |
| **Std_39000/1** | 18.74 | 18.79 | 0.04 | 39000 |  |  |
| **Std_39000/2** | 18.81 | 18.79 | 0.04 | 39000 |  |  |
| **Std_39000/3** | 18.82 | 18.79 | 0.04 | 39000 |  |  |
| **Std_3900/1** | 22.29 | 22.27 | 0.02 | 3900 |  |  |
| **Std_3900/2** | 22.25 | 22.27 | 0.02 | 3900 |  |  |
| **Std_3900/3** | 22.27 | 22.27 | 0.02 | 3900 |  |  |
| **Std_390/1** | 25.49 | 25.43 | 0.05 | 390 |  |  |
| **Std_390/2** | 25.39 | 25.43 | 0.05 | 390 |  |  |
| **Std_390/3** | 25.41 | 25.43 | 0.05 | 390 |  |  |
| **Std_39/1** | 29.13 | 28.78 | 0.32 | 39 |  |  |
| **Std_39/2** | 28.51 | 28.78 | 0.32 | 39 |  |  |
| **Std_39/3** | 28.69 | 28.78 | 0.32 | 39 |  |  |
| **n** | / |  |  | / |  |  |

**E:** Real time qPCR of *nirK* gene for OTU4 and OTU5

| **Name** | **Ct SYBR** | **Ct Mean SYBR** | **Ct Dev. SYBR** | **Amount SYBR [Copies]** | **Amount Mean SYBR** | **Amount Dev. SYBR** |
| --- | --- | --- | --- | --- | --- | --- |
| **Std_2.8E6/1** | 12.28 | 12.22 | 0.12 | 2.80E+06 |  |  |
| **Std_2.8E6/2** | 12.08 | 12.22 | 0.12 | 2.80E+06 |  |  |
| **Std_2.8E6/3** | 12.3 | 12.22 | 0.12 | 2.80E+06 |  |  |
| **4/1** | 20.8 | 20.92 | 0.3 | 6528 | 6100 | 1214 |
| **4/2** | 21.27 | 20.92 | 0.3 | 4731 | 6100 | 1214 |
| **4/3** | 20.69 | 20.92 | 0.3 | 7043 | 6100 | 1214 |
| **Std_280000/1** | 15.51 | 15.44 | 0.09 | 2.80E+05 |  |  |
| **Std_280000/2** | 15.47 | 15.44 | 0.09 | 2.80E+05 |  |  |
| **Std_280000/3** | 15.34 | 15.44 | 0.09 | 2.80E+05 |  |  |
| **6/1** | 19.31 | 19.53 | 0.19 | 18394 | 15841 | 2212 |
| **6/2** | 19.64 | 19.53 | 0.19 | 14655 | 15841 | 2212 |
| **6/3** | 19.66 | 19.53 | 0.19 | 14475 | 15841 | 2212 |
| **Std_28000/1** | 18.61 | 18.59 | 0.03 | 28000 |  |  |
| **Std_28000/2** | 18.61 | 18.59 | 0.03 | 28000 |  |  |
| **Std_28000/3** | 18.55 | 18.59 | 0.03 | 28000 |  |  |
| **Std_2800/1** | 22.01 | 21.79 | 0.31 | 2800 |  |  |
| **Std_2800/2** | 21.43 | 21.79 | 0.31 | 2800 |  |  |
| **Std_2800/3** | 21.93 | 21.79 | 0.31 | 2800 |  |  |
| **Std_280/1** | 25.17 | 25.19 | 0.02 | 280 |  |  |
| **Std_280/2** | 25.22 | 25.19 | 0.02 | 280 |  |  |
| **Std_280/3** | 25.19 | 25.19 | 0.02 | 280 |  |  |
| **Std_28/1** | 28.8 | 28.96 | 0.26 | 28 |  |  |
| **Std_28/2** | 29.26 | 28.96 | 0.26 | 28 |  |  |
| **Std_28/3** | 28.81 | 28.96 | 0.26 | 28 |  |  |
| **n** | / |  |  | / |  |  |

Note: *Sponge: 1 is *A. queenslandica* from the Linshui port; 2 is *S. vesparium* from the Linshui port; 3 is *Iotrochota* sp. from Yongxing Island; 4 is *X. testudinaria* from Yongxing Island; 5 is *C. australiensis* sp. from Yongxing Island; 6 is *Cinachyrella* sp.from Yongxing Island. “n” means negative control.
